# Assessment of Immature Granulocytes Percentage to Predict Severe Bacterial Infection in Latvian Children: An Analysis of Secondary Data

**DOI:** 10.3390/medicina54040056

**Published:** 2018-08-10

**Authors:** Jana Pavare, Ilze Grope, Dace Gardovska

**Affiliations:** Department of Pediatrics, Riga Stradins University, Dzirciema street 16, Riga, LV 1007, Latvia; ilze.grope@rsu.lv (I.G.); dace.gardovska@rsu.lv (D.G.)

**Keywords:** bacterial infections, sensitivity, specificity, immature granulocytes, Latvia, child, sepsis

## Abstract

*Background*: Detection of small proportion of serious bacterial infections (SBI) with a potentially life threating course in a large group of children with fever admitted to emergency department (ED) is still complicated. Measurement of immature granulocytes (IG) percentage may be used as a marker of bacterial infections. The aim of the study was to evaluate whether the IG percentage is a useful additional predictive marker of SBI. *Methods*: This study included 258 children with febrile infections that were admitted to the ED. Clinical follow-up, microbiological and radiological tests were used as reference standards for the definition of SBI. Study population was categorized into two groups: (1) infected patients with no suspicion of SBI (*n* = 75); (2) patients with suspicion of SBI (*n* = 183). IG percentage, white blood cell count (WBC), and C-reactive protein (CRP) levels were analyzed from the first routine blood samples at hospital admission. *Results*: A statistically significant difference in IG percentage levels was observed in children with SBI and those without—the mean IG percentage was 1.2% for the SBI group, 0.3% for those without SBI. The cutoff level of IG percentage to predict SBI was 0.45 (84% specificity, 66% sensitivity, 90% positive predictive value). We combine variables and evaluate their additive values. The sensitivity of WBC to detected SBI improved from 74% to 85% when IG percentage was added to the prediction models. When CRP, WBC, and IG percentage were combined, the sensitivity to predict SBI increased to 93%, the specificity to 86% (95% CI 77%–93%). Receiver operator characteristic analysis to predict SBI showed an area under the curve (AUC) of 0.80 for IG percentage. *Conclusion*: Addition of IG percentage to traditionally used markers of SBI as WBC and CRP may help to identify children with serious bacterial infections. Furthermore, IG percentage can be rapidly obtained from the traditional full blood count without any extra sampling and costs.

## 1. Introduction

Fever is one of the most common causes for admittance of children to the emergency department. Most children suffering from fever have simple self-limiting viral infections, however a small proportion will develop serious bacterial infections that are potentially life-threating. The low prevalence of serious illness and the frequent presence of pediatric patients in emergency department at the early stage of the illness, when severity of disease is still not apparent, pose for early and accurate diagnosis of serious infections a challenge [1,2,3,4]. To support the decision making in febrile children, a lot of clinical and laboratory prediction models have been investigated, but the interpretation and application process of these models in clinical practice is still complex [2,3,4,5,6].

Nowadays, modern automated hematology analyzers are available that can measure additional infection parameters, such as immature granulocytes (IG), which may be used as markers of serious bacterial infections [7,8]. However, despite availability of IG measurement, it has not yet been incorporated in the routine diagnostic tool for patients with infection. New generation analyzers can now provide automated, very precisely enumerated actual IG count and percentage in peripheral blood samples [8,9,10]. Several recent studies have investigated the role of IG percentage measurement as a potential marker to predict severity of an infection [11,12,13,14]. However, these studies were mainly focused on critically ill adult patients at intensive care units. Only one study investigated IG in a common outpatient clinical setting covering all age groups, including pediatric, obstetrics, and geriatric population [15]. Moreover, the performance of IG measurement compared with conventional infection markers, such as white blood cell count (WBC), absolute neutrophil count (ANC), and C-reactive protein (CRP) remains unclear [9,12,13].

Thus, the aim of this study was to evaluate whether the measurement of IG percentage is a useful predictive marker of serious bacterial infections (SBI) when compared with conventional infection markers such as WBC, CRP, and ANC in pediatric patients.

## 2. Materials and Methods

### 2.1. Study Population

The population of this retrospective study consisted of patients admitted due to infection to the emergency department of the Children’s clinical University hospital in Riga, Latvia during June 2015 and December 2016. The University hospital is the only tertiary level children hospital in the country with around 64,000 pediatric emergency department visits annually.

The inclusion criteria for the study were suspected diagnosis of any type of infection and age between one month and 18 years. Exclusion criteria were antibacterial therapy within the last 48 h, vaccination within five days before the start of illness, any immunodeficiency disease, malignancies, congenital metabolic defects, and other diseases that may alter the levels of inflammatory markers.

A total of 258 patients fulfilled the entry criteria and were selected for data analysis. Patients were classified into two groups: (I) infected patients without serious infection (*n* = 75) and (II) patients with SBI (*n* = 183).

### 2.2. Clinical Protocol and Definitions

Infection severity was defined at the emergency department. Within two to five days, two experienced clinicians revised whether infection severity was evaluated correctly at the initial moment of diagnosis to ensure correct classification of the study participants. SBI at emergency department and during the later revision of clinicians was defined based on available clinical, imaging, and later also on microbiological data as having either bacteremia, pneumonia (radiographically confirmed), meningitis, osteomyelitis, intra-abdominal infection, complicated urinary tract infection, skin/soft tissue infection, culture positivity of usually sterile body fluid, or diagnosis by radiology (pneumonia, osteomyelitis, intra-abdominal infection) [16]. The group of infected patients with fever, but without SBI included in most cases children with upper and lower respiratory tract infections of viral origin and patients with acute gastroenteritis mainly of rotavirus, norovirus, or adenovirus.

### 2.3. Data Collection and Laboratory Assays

The study parameters were collected during the first routine blood analysis sampling at the emergency department of patients with a suspicion of infection. Blood samples were obtained by venipuncture in EDTA vacutainer tubes. Blood cultures were collected by two separate vein punctures for all patients with suspicion of sepsis.

The IG percentage was calculated with an automated hematology analyzer Sysmex XE 2100. The IG measurement that includes promyelocytes, myelocytes, and metamyelocytes was performed in the differential channel of the Sysmex XE 2100. A specific lysing reagent causes mature WBC membranes disruption, leaving bare nuclei, but immature myeloid cells remain intact due to low cell membrane lipid content. The increased permeability of leucocytes allows for a polymethine dye to enter the cells with high affinity for nuclei acid. The cells are analysed by nucleic acid fluorescence and side scatter.

The IG percentage is defined as the percentage of the total WBC count [10,12]. CRP levels were measured by the latex method (Cobas Integra; Roche Diagnostics, Risch-Rotkreuz, Switzerland), the lowest assay sensitivity being 0.085 mg/L.

### 2.4. Ethical Considerations

This study used secondary, re-identified data and has been approved by the Medical Ethics Committee of the Riga Stradins University (No. 4/28.05.2015). All of the patients included in the study received the standard care according to the hospital guidelines.

### 2.5. Statistical Analyses

The data were analysed while using IBM SPSS version 22.0. Descriptive statistics were done to identify the characteristics of the categorical and continuous variables. Categorical variables are presented as numbers and percentages. The Kolmogorov-Smirnov test was used to check whether the continuous variables followed normal distribution. As the biomarkers did not follow a normal distribution, the medians and interquartile ranges are presented. Differences of continuous between groups were compared using the Mann-Whitney test. Collinearity diagnostics were utilized to evaluate the correlation amongst the variables. Unadjusted and adjusted logistic regression models were used to find the best predictors of serious bacterial infections, according to WBC, IG percentage, and CRP. Odds ratios (OR and their corresponding 95% confidence intervals are presented. The goodness-of-fit of the logistics regression models was assessed by the Hosmer-Lemeshow test. Furthermore, The Nagelkerke’s *R*^2^ was used as measure for the completeness of each studied model. Both tests, the Hosmer-Lemeshow and the Nagelkerke’s R^2^, were used to evaluate whether the association between the independent variables and the outcome variables were linear. In a second step, receiver-operating characteristic (ROC) curves were developed for each of the continuous biomarkers presenting the area under the curve, including the 95% CI for each biomarker. The Youden’s index was used to determine the best cut-off values for each indicator to maximize both, sensitivity and specificity (maximum+ = sensitivity + specificity − 1). A two-tailed *p* value < 0.05 was considered to be statistically significant.

## 3. Results

The baseline characteristics of the study population are presented in Table 1. The mean age of infected patients without serious bacterial infection was 60.3 months, and 76.6 months for the SBI patients, respectively. The prevalence of being male was 42% in patients with and 48% without SBI. The majority of cases with SBI were due to intra-abdominal infections, nephritis, or lower respiratory tract infections (69% of all SBI infections). Bacteremia confirmed by two separate positive blood cultures was detected in 32 SBI patients (17%). The most common detected bacteria in blood steam were *Staphylococcus aureus* (12 patients), *Escherichia coli* (eight patients), and *Streptococcus pneumoniae* (four patients). *Neisseria meningitidis* was identified both from blood culture and cerebrospinal fluid in three patients. The upper and lower respiratory tract viral infections, and Rotavirus and Norovirus infections were the main causes of infection in patients without SBI.

IG percentage, CRP, ANC, and WBC were all significantly higher in SBI patients when compared with those without severe infection.

**Figure 1:**
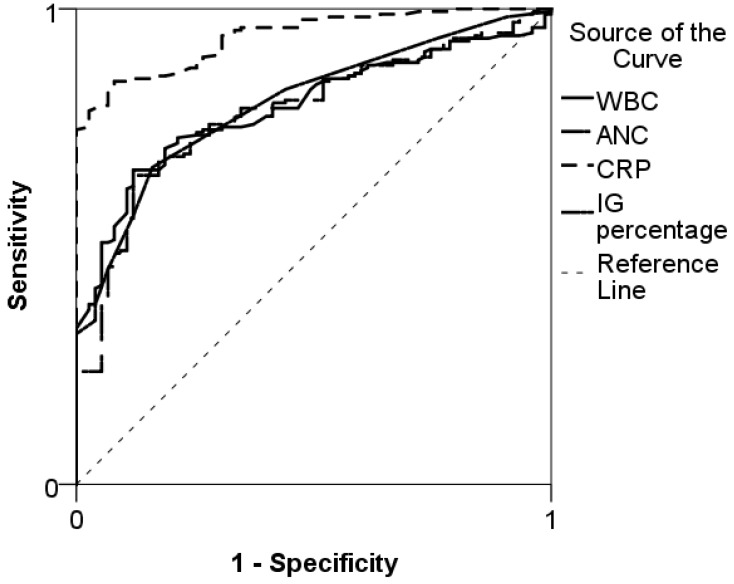
Receiver operating curves for immature granulocyte (IG) percentage, white blood count (WBC), absolute neutrophil count (ANC), and C-reactive protein (CRP) in predicting serious bacterial infections in children.

The area under the curve (AUC) of IG percentage to predict SBI was 0.80 (95% confidence interval (CI) 0.74–0.85) (Figure 1). The corresponding AUC for CRP was 0.94 (95% CI 0.91–0.96), for ANC 0.78 (95% CI 0.72–0.84) and for WBC 0.79 (95% CI 0.73–0.84).

Table 2 shows the sensitivity, specificity, and predictive values for the best cut-off value identified of the different markers of serious bacterial infections in the study population. The sensitivity for the best cut-off level for IG percentage (66%) was lower when compared to CRP, ANC, or WBC. However, in regards to specificity and positive predictive value IG percentage performed better than ANC or WBC. CRP, when using an optimal cut-off level of 56.5 mg/L showed the best diagnostic test performance. We combine variables and evaluate their additive values. The sensitivity of WBC to detected SBI improved from 74% to 85% when IG percentage was added to the prediction models and to 92% (95% CI 88%–96%) when CRP and WBC were measured. Finally, when CRP, WBC, and IG percentage were combined, the sensitivity to predict SBI increased to 93% (95% CI 88%–96%) and the specificity to 86% (95% CI 77%–93%).

Table 3 presents the odds ratios (OR) of the different biomarkers to predict SBI in children using different models. An increase in one unit of WBC was associated with a 1.23-fold increased risk (95% CI 1.15–1.32) of SBI and an increase in one mg/L of CRP resulted in a 1.07-fold increased risk of SBI (95% CI 0.05–1.1) in children. IG percentage was statistically significantly associated with an increased likelihood of SBI per one percent increase in IG (3.40-fold increased risk (95% CI 1.65–5.15)). In the model containing all three markers (Model 5), the OR for WBC increased from 1.23 to 1.31 as compared with Model 1 (only WBC included), whereas OR for CRP increased from 1.07 (Model 2, only CRP included) to 2.24 (Model 5). Adding IG% to the model (Model 5) increased the OR of WBC and CRP, whereas the OR for IG% decreased when compared with Model 3 where only IG% was used. Finally, the best biomarker to predict SBI in terms of Nagelkerke’s *R*^2^ was CRP (44%). The combination of WBC and CRP increased *R*^2^ to 72%; and the use of all three markers together (WBC, CRP, IG percentage) showed the highest *R*^2^ (79%).

## 4. Discussion

Our study found that IG percentage is a useful marker to predict the severity of infection and it adds information to the conventional infection markers WBC, ANC, and CRP for the early identification of pediatric patients with SBI. Specifically, when IG percentage was used together with CRP and WBC nine out of 10 children with SBI were correctly identified.

The prevalence of SBI in the children population is still high and the fast identification of patients with potentially serious and life threating conditions is important in the clinical setting. Elevated count of IG in the peripheral blood indicates increased bone marrow activity as the response to bacterial infection [17]. Adult patients with clinically apparent bacterial infection showed significantly higher numbers of myeloid progenitor cells when compared with healthy controls [17].

Whereas, most of studies investigating IG as a marker of SBI were performed on seriously ill adult patients or neonatal patients [10,12,13,17,18], only a few studies included not seriously ill patients [9,10,11,15]. Furthermore, information about IG percentage in children population is scant [9,15]. Our study added valuable information on the performance of IG percentage in children with different grades of infections directly of all age-group. Our results are in line with those of previous studies, revealing an association between a higher IG percentage in patients and positive blood bacterial culture [9,10,12]. Moreover, patients with suspected septicemia and positive blood culture had higher level of IG percentage than those with negative blood culture [10,12]. Furthermore, it was found that IG percentage in blood culture positive children patients were significantly higher than in culture negative patients [9].

In the study of Nierhaus et al., IG percentage showed the highest discriminative value for infection in the first 48 h in surgical intensive care patients. Interestingly, in contrast to CRP, only IG percentage was a significant predictor of severe infection [13]. Current evidence suggests that IG percentage may perform better in the early diagnosis of severe infection than for instance CRP or WBC [12,19].

To increase the accuracy of prediction of severe infections the combination of IG with other markers may be used [9,10,12,19,20,21,22]. Past research, in agreement with our results, have revealed that the predictive value of IG percentage in critically ill adult patients improves for each day when IG percentage is added to WBC and CRP [12]. Moreover, the IG percentage adds to WBC and CRP in the early exclusion of infection and can be obtained routinely without extra blood sampling or costs [12]. When considering that CRP usually starts to increase after 24 h of infection start, measuring WBC and IG percentage may help to identify children with SBI at a very early stage helping clinical decision makers to start treatment accordingly.

In our study, the best sensitivity and specificity was obtained while using a threshold of 0.45 for IG percentage in children. Previous research have revealed optimal cut-off levels for IG percentage varying between 0.4–0.5 with sensitivities varying between 58%–96% and specificity ranging from 76%–80% [9,10,12,15]. Thus, which cut-off level to be used within that particular range may depend on the local circumstances. Pooling data of existing research projects may offer a better estimate of the ideal threshold to be used in children to diagnose SBI.

In our study, we found the highest sensitivity 93% (95% CI 88%–96%) when CRP, WBC, and IG percentage were combined, and the second highest specificity for above mentioned combination—86% (95% CI 77%–93%). We prefer to optimize sensitivity over specificity to identify more potential SBI patients, while considering that rule-out diagnostic tool in a typical emergency department (ED) setting with low prevalence of SBI is more reasonable and may further reduce the false negative rate.

The cut-off level detected for CRP in our study was 56.5 mg/L, which is comparatively low for the detecting of bacterial infections. At the same time, there in a systemic review and meta-analysis perform by Yo et al., the cut-off level for CRP for the detection of serious bacterial infections in children presenting with fever was 40 mg/L [6]. In the study performed by our research group some years ago we detected cut-off level of 99.5 mg/L for CRP to identify patients with bacteremia (sensitivity of 80.6% and specificity of 82.1%) [23]. Further studies in children population are desirable to discuss the overall diagnostic accuracy of CRP.

Naturally, our study had some limitations. For instance, our study is single center, retrospective design, we did not include a control group consisting of healthy children in our study that may give valuable insight into the pattern of inflammatory markers in children without SBI. We had high proportion of gastroenteritis cases in patients group without SBI, which could be explained by low oral rehydration traditions in parents’ population of our country and therefore could influence the external validity of our study. However, as our study did not aim at obtaining population estimates, but testing a screening tool, the issue of external validity can be neglected. As well as not all children with SBI admitted to the emergency department of our hospital were included due to a small size of our research team and due to the approbation of new analyzer Sysmex XE 2100. Furthermore, our study focused on early laboratory diagnosis of patients with SBI and was not able to include information on any genetic influences or cellular abnormalities as potential triggers for the infection progress to a life threatening state.

## 5. Conclusions

Our findings suggest that SBI in children is associated with an increase in IG percentage. The IG percentage differs between patients with non-SBI and those with SBI. Furthermore, IG percentage adds to WBC and CRP in the early detection of serious bacterial infections in pediatric population and provides the additional diagnostic tool for physicians in identifying of a small proportion of high risk children in very intensive flow of patients at emergency department. IG percentage can be rapidly obtained from the traditional full blood count without any extra sampling and costs.

## Figures and Tables

**Table 1:** Baseline characteristics, infection focus and markers of the study population, according to severity of infection.

Characteristics of the Study Sample	Patients Without SBI (*n* = 75)	SBI Patients (*n* = 183)
**Baseline Characteristics**		
Boys, *n* (%)	36 (48)	78 (42)
Age, median (IQR), months	37 (16–96)	63 (13–140)
Length of hospitalization, median (IQR), days	3 (1–5)	10 (5–14)
**Infection Focus**, *n* (%)		
Intra-abdominal	0 (0)	44 (24)
Nephritis/pyelonephritis	0 (0)	42 (23)
Lower respiratory tract	12 (16)	36 (20)
Osteomyelitis	0 (0)	21 (11)
Meningitis	0 (0)	11 (7)
Occult bacteremia	0 (0)	10 (5)
Skin/soft tissue infection	0 (0)	10(5)
Upper respiratory tract	11 (15)	5 (3)
Toxic shock syndrome	0 (0)	2 (1)
Pericarditis	0 (0)	1 (0.5)
Gastroenteritis	52 (69)	1 (0.5)
**Infection Markers**		
IG percentage	0.3 (0.25–0.40)	0.6 (0.4–1.2)
CRP, median (IQR), mg/L	6 (1–23)	106 (56–200)
ANC, median (IQR), ×10^9^/L	7.1 (4.5–8.6)	12.2 (12.2–16.8)
WBC, median (IQR), ×10^9^/L	9.9 (7.5–12.0)	17 (11.6–22.9)

IQR, interquartile range; SBI, serious bacterial infection; IG, immature granulocytes; CRP, C-reactive protein; ANC, absolute neutrophil count; WBC, white blood cell count.

**Table 2:** Sensitivity, specificity, and predictive values for different markers and their combinations of serious bacterial infections in children.

Variable	Cutoff Value	Sensitivity % (95% CI)	Specificity % (95% CI)	PPV % (95% CI)	NPV % (95% CI)
IG percentage	0.45	66 (59–73)	84 (73–91)	90 (84–95)	51 (42–60)
CRP, mg/L	56.5	75 (68–81)	100 (95–100)	100 (97–100)	62 (53–71)
ANC, ×10^9^/L	8.6	73 (65–79)	73 (61–82)	86 (80–91)	52 (42–62)
WBC, ×10^9^/L	11.75	74 (67–80)	73 (61–82)	54 (80–91)	87 (44–64)
WBC and IG percentage	NA	85 (79–90)	64 (52–74)	85 (78–90)	64 (52–75)
WBC and CRP	NA	92 (88–96)	85 (74–92)	92 (86–94)	83 (73–90)
WBC, CRP and IG percentage	NA	93 (88–96)	86 (77–93)	94 (89–97)	84 (74–91)

PPV, positive predictive value; NPV, negative predictive value; CI, confidence interval; IG, immature granulocytes; CRP, C-reactive protein; ANC, absolute neutrophil count; WBC, white blood cell count; NA, not applicable.

**Table 3:** Logistic regression coefficients of different biomarkers to predict serious bacterial infections in children.

	Variables Included	WBC		CRP		IG %	
		OR	(95% CI)	OR	(95% CI)	OR	(95% CI)
Model 1	WBC	1.23	(1.15–1.32)	NA	NA	NA	NA
Model 2	CRP	NA	NA	1.07	(1.05–1.1)	NA	NA
Model 3	IG%	NA	NA	NA	NA	3.40	(1.65–5.15)
Model 4	WBC, CRP	1.23	(1.10–1.37)	1.07	(1.04–1.12)	NA	NA
Model 5	WBC, CRP, IG%	1.31	(1.14–1.49)	2.24	(1.20–3.38)	1.08	(1.05–1.11)

CI, confidence interval; WBC, white blood cell count; NA, not applicable; CRP, C-reactive protein; IG, immature granulocytes.
